# Influence of Cooling Rate During β Annealing on the Microstructure and Properties of Ti55531 Titanium Alloy

**DOI:** 10.3390/ma19081486

**Published:** 2026-04-09

**Authors:** Xiaoyuan Yuan, Shun Han, Yuxian Cao, Leilei Li, Xinyang Li, Ruming Geng, Simin Lei, Jianguo Wang, Chunxu Wang, Yong Li

**Affiliations:** 1 Institute of Special Steels, Central Iron and Steel Research Institute Company Limited, Beijing 100081, China; 18810865090@163.com (X.Y.); cyx726924@163.com (Y.C.); 18810296519@163.com (L.L.); lixinyang@nercast.com (X.L.); gengruming@nercast.com (R.G.); leisimin@nercast.com (S.L.); liyong@nercast.com (Y.L.); 2 State Key Laboratory of Advanced Special Steel, Beijing 100081, China; 3 School of Materials Science and Engineering, Northwestern Polytechnical University, Xi’an 710072, China; jianguow@nwpu.edu.cn

**Keywords:** Ti55531 titanium alloy, cooling rate, mechanical properties, Widmanstätten structure, crack extension

## Abstract

As a high-performance lightweight structural material with superior strength, Ti55531 titanium alloy has been widely adopted in critical load-bearing components such as landing gears and airframe frames in the aerospace sector to achieve significant weight reduction. However, when the tensile strength of Ti55531 exceeds 1250 MPa, the fracture toughness typically falls below 50 MPa·m^1/2^. In this study, we addressed this challenge by precisely controlling the cooling rate during β annealing heat treatment. Through careful regulation of the cooling rate from the high-temperature β phase region to the aging temperature region, the Widmanstätten structure was successfully introduced into the Ti55531 titanium alloy. The experimental results demonstrate that this microstructure achieves a high tensile strength of 1252 MPa at a cooling rate of 2.5 °C/min, while simultaneously improving the elongation and fracture toughness to 9% and 84 MPa·m^1/2^, respectively. Microstructural analysis reveals that the basket-weave structure plays a crucial role in maintaining high strength. Meanwhile, the Widmanstätten structure effectively increases the energy required for crack extension by resisting crack propagation and altering the crack propagation path, thus significantly enhancing fracture toughness. These findings offer a promising pathway for overcoming the traditional trade-off between strength and toughness in high-performance titanium alloys.

## 1. Introduction

Titanium alloys have become indispensable materials in critical sectors, including aerospace, defense, biomedical engineering, and energy-related chemical industries, owing to their exceptional specific strength, outstanding corrosion resistance, and favorable biocompatibility [1,2,3,4,5]. Statistical data indicate that titanium alloys constitute approximately 4.5–14% of the structural weight in modern civil aircraft, with this proportion escalating to 20–41% in the latest generation of fighter aircraft [6,7]. Among the diverse titanium alloy systems, metastable β-type titanium alloys are particularly well-suited for the fabrication of aircraft load-bearing components, attributed to their superior strength–toughness synergy [8,9,10]. Ti-5Al-5V-5Mo-3Cr-1Zr (hereafter referred to as Ti55531) represents the novel generation of a high-strength and high-toughness titanium alloy [11,12,13,14,15] co-developed by Russian VSMPO-AVISMA and European Airbus. Ti55531 exhibits a comprehensive array of advantageous properties, including insensitivity to segregation, high strength, high toughness, a wide processing window, and excellent hardenability [16,17,18]. Consequently, it is highly suitable for manufacturing structural components subjected to high-stress conditions. Currently recognized as one of the latest international high-strength, high-toughness titanium alloys, Ti55531 has been successfully implemented in critical load-bearing applications, such as the main landing gear support beam of the Airbus A380 [14,19].

Currently, the heat treatment processes for Ti55531 mainly involve solution treatment followed by aging [20,21,22,23,24]. The mechanical properties of Ti55531 alloy are critically dependent on the control of α phase morphology, specifically the equiaxed primary α phase and fine secondary acicular α phase, which play a pivotal role in balancing strength and toughness [25,26,27]. However, a fundamental trade-off between strength and fracture toughness exists, i.e., when the tensile strength exceeds 1250 MPa, the fracture toughness typically falls below 50 MPa·m^1/2^ [28,29,30]. This limitation arises because the fine secondary acicular α phase cannot effectively impede crack propagation, while the coarse equiaxed α phase detrimentally affects strength enhancement. In contrast, the Widmanstätten structure (Ws), characterized by regularly arranged elongated lamellar α and β phases, has been reported to improve ductility with only a limited reduction in strength [31,32]. This microstructure typically forms during processing or annealing in the β phase region, followed by slow cooling [33,34,35]. Nevertheless, comprehensive investigations regarding the impact of Ws on the mechanical properties of high-strength titanium alloys (with strength ≥ 1100 MPa) remain scarce.

Existing research on Ti55531 titanium alloy has predominantly focused on its hot working behavior and solution-aging treatment parameters [19,22,36,37,38], with significant gaps in understanding the β annealing processes. It is particularly noteworthy that the mechanical properties of Ti55531 are highly sensitive to the cooling rate [10,34], for which the underlying mechanisms remain insufficiently elucidated. To address this knowledge gap, this study systematically investigates the effects of varying cooling rates during β annealing on the microstructure and mechanical properties of extruded Ti55531 forgings. This investigation provides foundational theoretical insights for optimizing the heat treatment process of integral extrusion components made from high-strength Ti55531 titanium alloy.

## 2. Materials and Methods

**Figure 1:**
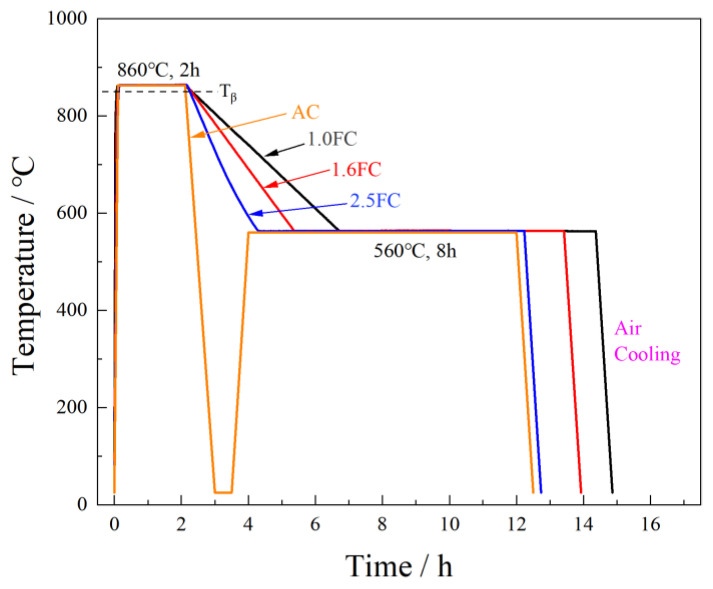
Schematic illustration of the heat treatments.

A Φ500 mm ingot of Ti55531 titanium alloy was produced by pressing electrodes using sponge Ti, sponge Zr, high-purity Al, and corresponding intermediate alloys (Mo40V40Al20, AlV65, AlCr70) as raw materials, followed by triple melting in an INTECO 10T vacuum consumable arc furnace (produced by INTECO melting and casting technologies GmbH, Bruck an der Mur, Austria). Melting was conducted under vacuum (≤2 Pa) with water-cooled copper mold solidification. The chemical composition of the ingot is presented in Table 1, and the β-transus temperature (T_β_) was determined to be 847 °C in accordance with the metallographic method specified in HB 6623.2. The as-cast Φ500 mm ingot was forged and drawn into Φ420 mm bars, which were then heated to 830 °C and extruded into thick-walled tubes with 52 mm wall thickness through single-pass hollow extrusion using a 6300-ton horizontal extrusion press. Subsequently, wire cutting was performed to obtain specimens for tensile and fracture toughness tests under different heat treatments. All the tested specimens were heated at 860 °C for 2 h, and then cooled at different cooling rates: (1) air cooling to room temperature followed by aging at 560 °C for 8 h (denoted as AC sample); (2) furnace cooling at controlled rates of 2.5 °C/min, 1.6 °C/min and 1.0 °C/min to 560 °C, followed by aging at 560 °C for 8 h (denoted as 2.5FC, 1.6FC and 1.0FC samples, respectively). Notably, the air-cooling rate is one order of magnitude higher than the furnace-cooling rate. After aging, the cooling mode for all the specimens was air cooling. The schematic illustration of the heat treatments is shown in Figure 1. Here, a brief explanation of the intermediate cooling step in the AC specimen is necessary. According to the literature, Ti55531 alloy exhibits high hardenability [39]. After air cooling from 860 °C to room temperature, its microstructure is predominantly composed of a β phase, with an α-phase content of only 0.086% [40]. It can be reasonably inferred that if the alloy is directly air-cooled from 860 °C to 560 °C, the resulting α-phase content would be even lower. Therefore, the influence of the intermediate cooling step in the AC sample on the final microstructure of the specimen can be considered negligible in this study.

Room-temperature tensile tests were performed on a Landmark series MTS-880 testing machine (MTS Systems Corporation, Eden Prairie, MN, USA), in accordance with GB/T 228.1-2021 [41]. The tensile specimens had dimensions of M12 mm × 65 mm, with a gauge section diameter of 5 mm and a gauge length of 25 mm. Room-temperature fracture toughness tests were performed on a Landmark series MTS-370 testing machine (MTS Systems Corporation, Eden Prairie, MN, USA), in accordance with GB/T 4161-2007 [42]. The fracture toughness tests were conducted using compact tension (C-T) specimens with dimensions of 62.5 mm × 60 mm × 25 mm. The notch direction was oriented perpendicular to the extrusion direction.

All specimens for X-ray diffraction (XRD) and scanning electron microscopy (SEM, Zeiss Supra 55, Carl Zeiss, Oberkochen, Germany) were prepared via electrolytic polishing. The samples were ground with a pre-grinder and sandpaper up to 2000# grit, followed by mechanical polishing. The mechanical polishing was performed using an electrolyte of 6% perchloric acid + 30% n-butyl alcohol + 64% methyl alcohol, with a direct voltage of 20 V and a current of approximately 1 mA at a temperature of around −20 °C for 20–25 s. XRD analysis was performed using a Bruker D8 Advance diffractometer (Bruker AXS GmbH, Karlsruhe, Germany) with Cu Kα radiation (λ = 1.5406 Å), operating at 40 kV and 40 mA. Scans were conducted from 30° to 80° (2θ) with a step size of 0.02°/s. The phase analysis was conducted using the method of XRD pattern matching against the powder diffraction file (PDF) database.

The specimens for electron backscattered diffraction (EBSD) analyses were prepared via vibration polishing. Prior to vibration polishing, the sample undergoes mechanical polishing in the same manner as SEM sample preparation. Vibration polishing was then performed on the mechanically polished sample using a colloidal silica suspension at a voltage of 190 V and a frequency of 135 Hz, for a duration of 2 h, until complete removal of surface stress marks was achieved.

For transmission electron microscopy (TEM, Tecnai G2 F30S-Twin (FEI Company, Hillsboro, OR, USA) operated at the electron accelerating voltage of 300 kV) observation, the specimens were initially cut into 0.3–0.5 mm thick slices by wire cutting. The specimens were sequentially pre-ground with 400#, 800#, 1000#, and 2000# sandpaper to reduce the thickness to 100 μm, and then punched into discs with a diameter of 3 mm. Further grinding with 2000# sandpaper reduced the thickness to 50 μm. Finally, the TEM samples were prepared by electrolytic double-jet thinning using the same electrolyte. The voltage was set to approximately 30 V, and the temperature of the double-jet solution was maintained between −30 °C and −20 °C.

## 3. Results

### 3.1. Phase Composition and Microstructure

**Figure 2:**
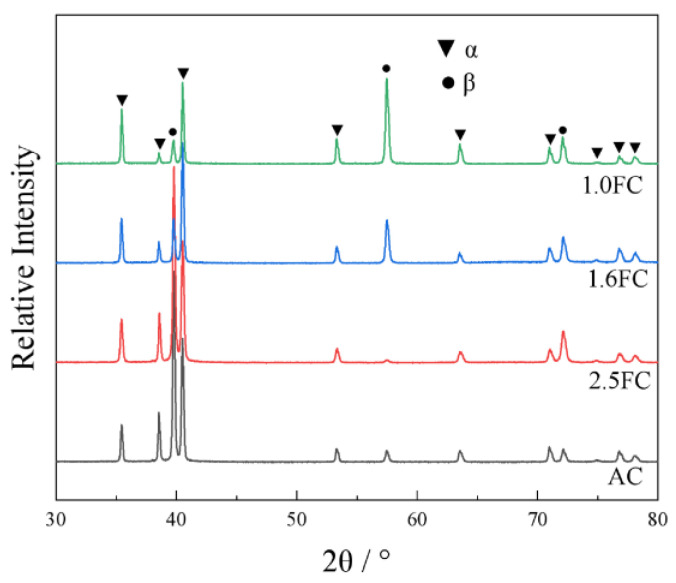
XRD patterns of the samples after heat treatment with different cooling rates.

Figure 2 presents the XRD patterns of samples subjected to different β annealing cooling rates. Peak identification reveals that all samples consist solely of a hexagonal close-packed (HCP) α phase and a body-centered cubic (BCC) β phase, with no detectable diffraction peaks from other secondary phases. This observation confirms that varying the cooling rate does not induce additional phase transformation or secondary phase precipitation.

Rietveld refinement was performed on each XRD pattern to quantify the lattice parameters and phase fractions, as shown in Table 2. The lattice parameters of the α phase maintain stable values of a = b = 2.929–2.930 Å and c = 4.675–4.677 Å, while the β phase retains its characteristic BCC structure with a = b = c = 3.208–3.210 Å, demonstrating that the cooling rate has a minimal influence on the crystal structure in Ti55531. Notably, a pronounced trend emerges in phase content evolution with cooling rate. As β annealing cooling rate decreases, the fraction of α phase increases progressively from 53.4 ± 1.0 wt% in the AC sample to 77.6 ± 1.0 wt% in the 1.0FC sample, while the content of β phase correspondingly declines from 46.6 ± 1.0 wt% to 22.4 ± 1.0 wt%. The shift in phase content ratio is directly attributable to the control of the β→α transformation by cooling rate, i.e., higher cooling rates suppress the transformation process, resulting in greater retention of the β phase in the AC sample. Furthermore, micro-strain analysis reveals another cooling-rate-dependent phenomenon. Both α and β phases exhibit gradually decreased micro-strain with reduced cooling rate. This reduction stems from enhanced dislocation and lattice distortion recovery at lower cooling rates, as the system is afforded more time for relaxation.

**Figure 3:**
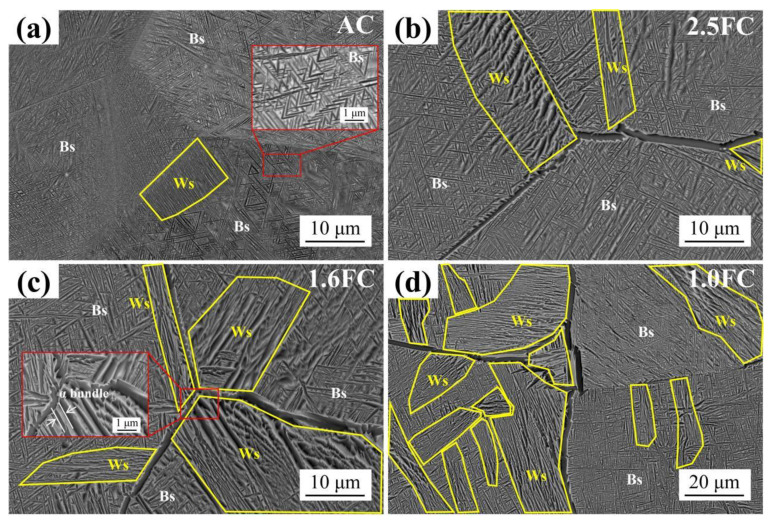
SEM patterns under different cooling rates: (**a**) air cooling, (**b**) 2.5 °C/min, (**c**) 1.6 °C/min, (**d**) 1.0 °C/min.

The SEM images of the samples subjected to varying β annealing cooling rates are shown in Figure 3. Under air-cooling conditions, the microstructure exhibits a characteristic Bs, comprising fine acicular α phase and β phase arranged in an interlaced and tightly woven manner, along with a small amount of Ws, as shown in Figure 3a. Notably, due to the suppression of α lamellae growth by rapid cooling, the α lamellae within the Ws of the AC sample are extremely fine, exhibiting a width comparable to that of the fine acicular α phase in Bs (<100 nm). A significant microstructural transition occurs when the cooling rate is reduced to 2.5 °C/min (Figure 3b). The grain boundaries (GBs) coarsen, appearing in a continuous or semi-continuous distribution. Concurrently, the Ws extends from the GB into the grain interior, accompanied by coarsening of the α lamellae. With a further reduction in cooling rate, the extended diffusion time enables more pronounced growth of Ws. The α lamellae become thicker and longer (Figure 3c), as the reduced cooling rate provides sufficient time for atomic diffusion. When the cooling rate is reduced to 1.0 °C/min, the Ws continues to grow, making contact and interlacing with each other (Figure 3d). Additionally, the acicular α phases in Bs also continue to coarsen with decreasing cooling rate. Incidentally, the α bundle composed of one α lamella and one β lamella is shown in the red box in Figure 3c, and its average width will be statistically analyzed subsequently.

Both Ws and Bs are composed of α and β phases. These two structures share identical crystal structures and chemical compositions, differing only in their morphological appearance. Consequently, conventional characterization techniques such as XRD and EBSD are inadequate for reliably distinguishing or quantifying the two structures. To address this limitation, the MIPAR software (version: 5.1.0) was employed to identify and differentiate Ws and Bs in the SEM images shown in Figure 3, and to calculate their respective fractions, as summarized in Table 3. The results indicate that the fraction of Ws increased from 7.2% in the AC sample to 46.3% in the 1.0FC sample, whereas the fraction of Bs decreased correspondingly from 92.8% to 53.7%. However, due to the intricately interwoven nature of the two structures, fully accurate identification by MIPAR could not be achieved. Therefore, the quantitative results presented here should be regarded as semi-quantitative.

**Figure 4:**
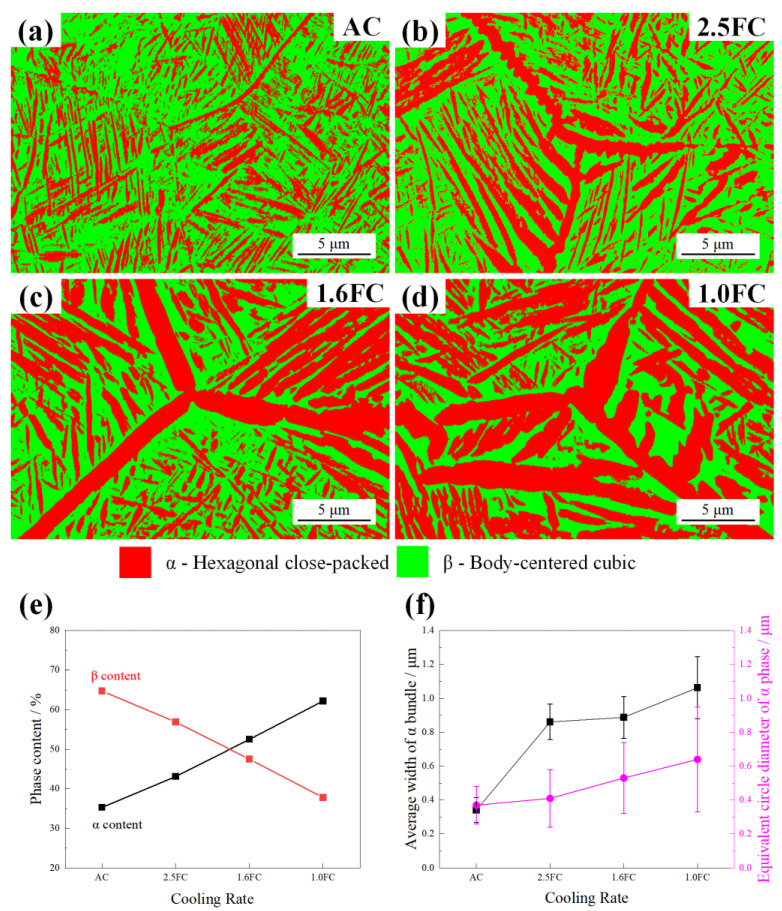
EBSD patterns under different cooling rates: (**a**) air cooling, (**b**) 2.5 °C/min, (**c**) 1.6 °C/min, (**d**) 1.0 °C/min, and the statistical phase content (**e**), as well as the average width of the α bundle and the equivalent circle diameter of the α phase (**f**).

Figure 4 presents the EBSD analysis of samples processed under different β annealing cooling rates. It can be clearly seen that the α phase, including α GBs, continues to coarsen with the decrease in cooling rate. Meanwhile, the Ws mainly grows and coarsens along the GBs into the grain interior. The AZtecCrystal software (version: 2.1) was used to quantitatively analyze the EBSD data. The volume fraction of the α phase increased from 35.3% in the AC sample to 62.2% in the 1.0FC sample, as shown in Figure 4e, exhibiting a trend consistent with prior XRD measurements. The equivalent circle diameter (ECD) of the α phase increased progressively from 0.37 ± 0.11 μm in the AC sample to 0.64 ± 0.31 μm in the 1.0FC sample, as shown in Figure 4f. Furthermore, the average width of the α bundle was quantified, which can be considered as an indirect statistic for the growth of Ws. The average width of the α bundle (calculated from at least 100 bundles obtained from three EBSD patterns per sample) keeps increasing from 0.34 ± 0.07 μm in the AC sample to 1.06 ± 0.18 μm in the 1.0FC sample with the decrement of cooling rate, as shown in Figure 4f.

**Figure 5:**
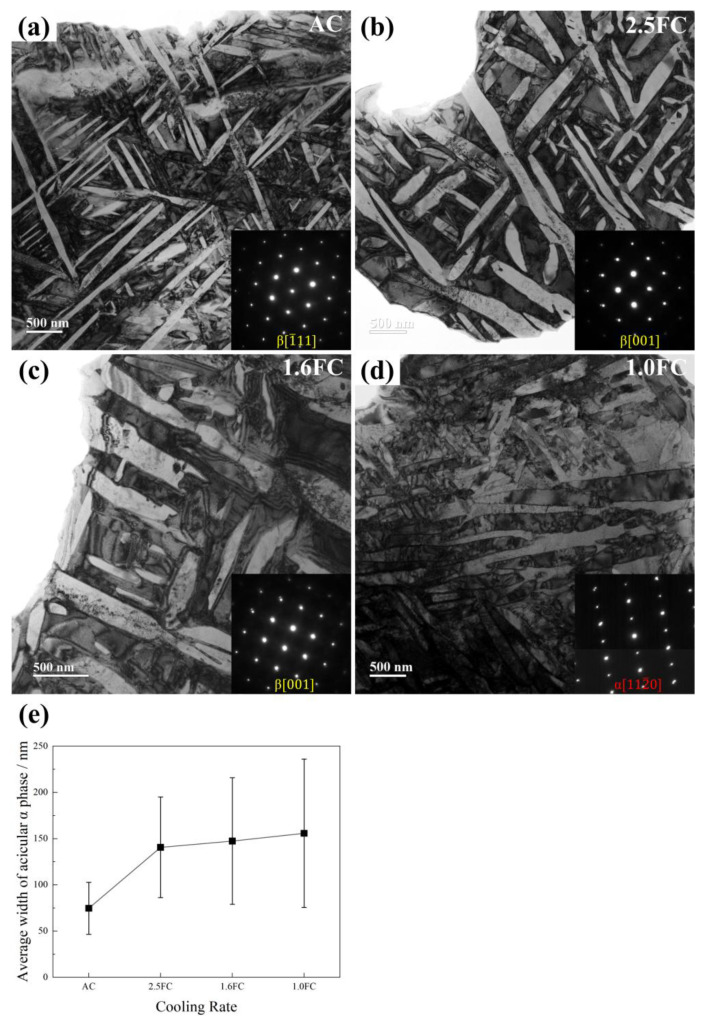
TEM patterns under different cooling rates: (**a**) air cooling, (**b**) 2.5 °C/min, (**c**) 1.6 °C/min, (**d**) 1.0 °C/min, as well as the statistical average width of acicular α phase (**e**).

Figure 5 presents the TEM images of samples subjected to different β annealing cooling rates. Under the [001] and [111] zone axes of the β phase, the α phase appears bright white (while under the [1120] zone axis of the α phase, it appears light gray-black) and is distributed in a crisscross pattern within the matrix. Indexing of the selected-area electron diffraction patterns confirms that only the α and β phases are present, with no other precipitates detected, which is consistent with the XRD results. Due to the relatively large scale of Ws and the limited observation area of TEM, only the width of the α phase within the Bs was measured from the TEM images, as summarized in Figure 5e. It should be noted that along the length direction, most α phases are connected end-to-end and interwoven, making their boundaries difficult to define accurately, whereas the boundaries in the width direction are distinct and allow reliable measurement. Furthermore, the TEM images clearly show that as the cooling rate decreases, the change in the width of the α phase becomes more pronounced. Therefore, statistical analysis of the α-phase width can reflect the influence of cooling rate on the microstructure. According to Figure 5e, as the cooling rate decreases, the width of the acicular α phase in Bs increases sharply from 74.6 ± 28.1 nm in the AC sample to 140.5 ± 54.5 nm in the 2.5FC sample, and then rises gradually to 155.7 ± 80.2 nm in the 1.0FC sample (based on width measurements of more than 200 acicular α phases taken from five TEM images per sample).

### 3.2. Mechanical Properties

**Figure 6:**
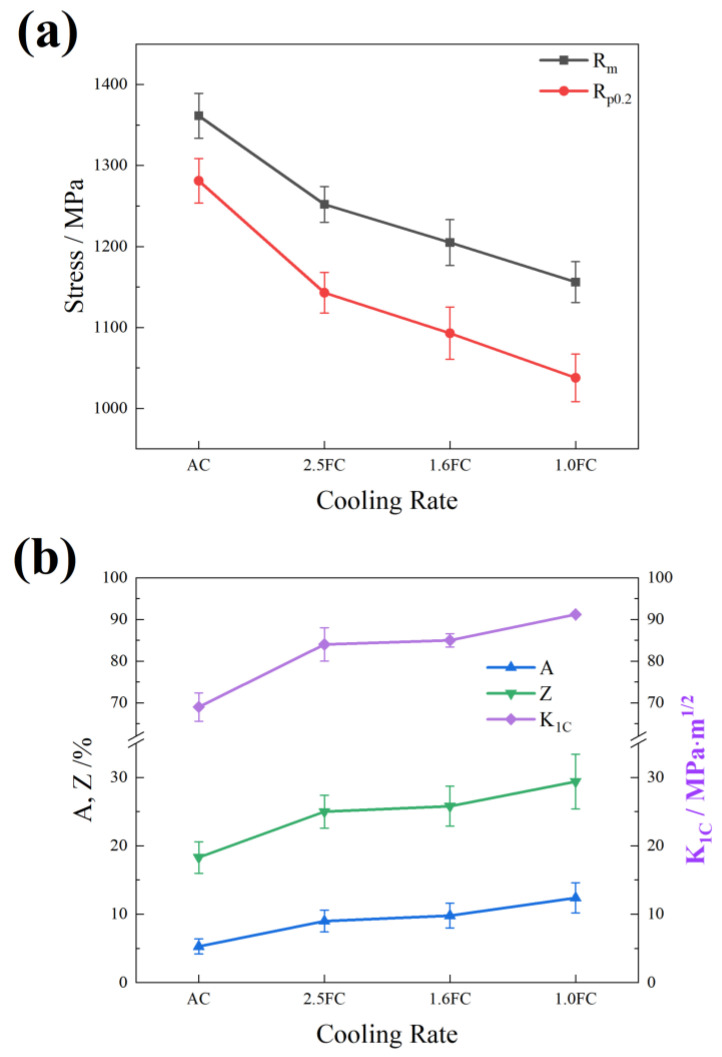
The mechanical properties under different cooling rates: (**a**) tensile strength and yield strength; (**b**) elongation after fracture, reduction of area, and fracture toughness.

The mechanical properties of Ti55531 titanium alloy under different cooling rates are presented in Figure 6. As the cooling rate decreases, the strength of the Ti55531 alloy shows a declining trend. The tensile strength decreases from 1361.4 ± 27.7 MPa for the AC sample to 1156.1 ± 25.2 MPa for the 1.0FC sample, while the yield strength drops from 1281.2 ± 27.5 MPa to 1037.9 ± 29.3 MPa. Conversely, the elongation and toughness improve with decreasing cooling rate. The elongation after fracture increases from 5.3 ± 1.1% for the AC sample to 12.4 ± 2.2% for the 1.0FC sample, the reduction in area improves from 18.3 ± 2.3% to 29.4 ± 4%, and the fracture toughness significantly increases from 69 ± 3.4 MPa·m^1/2^ to 91.2 ± 0.1 MPa·m^1/2^. These results clearly demonstrate the significant regulatory effect of cooling rate on the strength and fracture performance of Ti55531.

Notably, the sample cooled at 2.5 °C/min (2.5FC sample) demonstrates an excellent combination of high strength and toughness, with a tensile strength as high as 1252 ± 22.2 MPa, while maintaining an elongation of 9 ± 1.6% and a fracture toughness of 84 ± 4 MPa·m^1/2^. The most notable characteristic of this processing condition is the optimal balance between strength and toughness. This result holds significant importance for promoting the further application of Ti55531 titanium alloy in the aerospace field.

### 3.3. Fracture Surface Morphology

**Figure 7:**
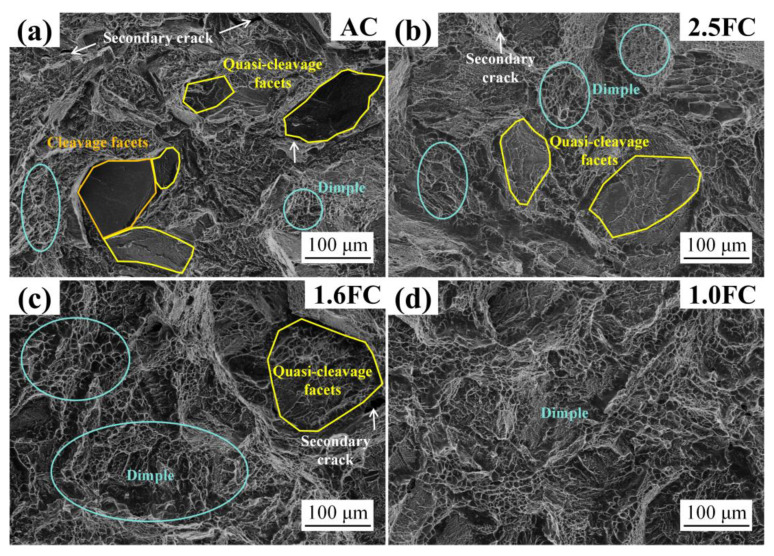
The fracture surface morphology of fracture toughness specimens under different cooling rates: (**a**) air cooling, (**b**) 2.5 °C/min, (**c**) 1.6 °C/min, (**d**) 1.0 °C/min.

The fracture surface morphology of fracture toughness specimens subjected to varying cooling rates is shown in Figure 7. At higher cooling rates (AC sample, Figure 7a), smooth cleavage facets, quasi-cleavage facets, a small number of dimples, and secondary cracks were observed on the fracture surface, which is characteristic of a brittle fracture, resulting in a relatively low fracture toughness value (only 69 ± 3.4 MPa·m^1/2^).

As the cooling rate decreases to 2.5 °C/min (2.5FC sample), the α-phase content in the alloy undergoes a significant increase (Figure 4e), accompanied by the growth and coarsening of Ws (Table 3 and Figure 4). Correspondingly, the fracture surface morphology shows complete disappearance of cleavage facets, a substantial reduction in quasi-cleavage features, and a corresponding increase in dimple count (Figure 7b). With further reduction in cooling rate, the content of α-phase continues to rise, the number of quasi-cleavage features diminishes further, and the dimple population increases progressively (Figure 7c).

When the cooling rate is reduced to 1.0 °C/min (1.0FC sample), the α-phase content in the material reaches 62.2%, with Ws undergoing additional growth and coarsening. Under these conditions, only dimples are exclusively observed in the fracture morphology, with no evidence of cleavage or quasi-cleavage facets (Figure 7d), yielding the highest fracture toughness of 91.2 ± 0.1 MPa·m^1/2^. This observation indicates that the fracture mode of the material has completely transitioned to a ductile fracture.

### 3.4. Crack Propagation

**Figure 8:**
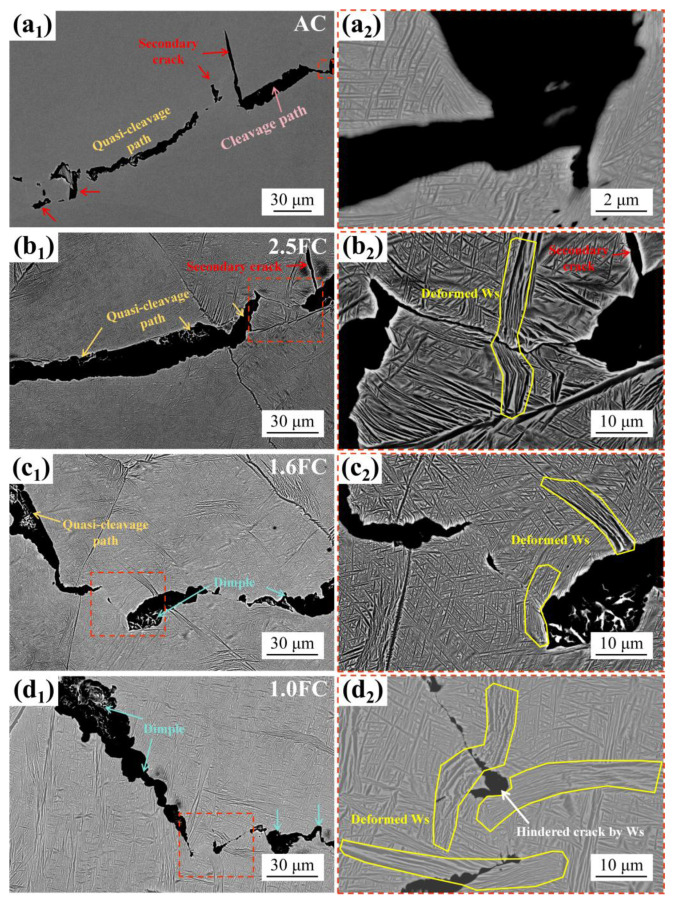
The crack propagation paths under different cooling rates: (**a_1_**) air cooling, (**b_1_**) 2.5 °C/min, (**c_1_**) 1.6 °C/min, (**d_1_**) 1.0 °C/min. Images (**a_2_**–**d_2_**) correspond to the enlarged views of the regions marked by red dashed boxes in (**a_1_**–**d_1_**), respectively.

To further investigate the influence of Ws on crack propagation, fracture toughness specimens subjected to different cooling rates were loaded to their maximum force (without fracture) using an MTS-370 testing machine. Following unloading, the resulting crack morphology was examined, as shown in Figure 8.

In the AC sample, crack propagation follows a relatively straight path, accompanied by numerous secondary cracks and distinct cleavage/quasi-cleavage features, as shown in Figure 8(a_1_), indicative of brittle fracture behavior. This morphology closely resembles the fracture surface shown in Figure 7a. Since the AC sample consists primarily of Bs with fine α-phase particles, this fine-grained morphology enhances yield strength via the Hall–Petch effect but does not effectively hinder crack propagation (Figure 8(a_2_)), ultimately resulting in a lower fracture toughness value.

When the cooling rate is reduced to 2.5 °C/min, the crack path exhibits noticeable deflection, as shown in Figure 8(b_1_), and both the number and length of the secondary cracks are significantly reduced compared to the AC sample. In the zigzag region of the crack, as shown in Figure 8(b_2_), the crack profile appears irregular. Furthermore, the crack traverses the Ws, inducing considerable plastic deformation within the Ws, whereas the Bs undergoes only minimal deformation.

With a further reduction in cooling rate to 1.6 °C/min, only a few relatively straight cracks are observed, as shown in Figure 8(c_1_). The crack profile becomes more irregular compared to that in the 2.5FC sample. Notably, the crack even displays discontinuous propagation, and within this region (Figure 8(c_2_)), deformed Ws is evident, suggesting that Ws can effectively resist crack propagation.

At the lowest cooling rate of 1.0 °C/min, the crack profile shows almost no straight segments, as shown in Figure 8(d_1_), and the crack propagation path is considerably elongated, reflecting clear ductile fracture characteristics. In regions of discontinuous crack growth (Figure 8(d_2_)), Ws with different orientations undergoes deformation, and the crack is even unable to penetrate the Ws directly, instead deviating by nearly 90 degrees.

Figure 8 collectively demonstrates that Ws promotes crack path tortuosity, increases the actual crack propagation length, and thereby raises the energy required for crack extension, which significantly enhances the fracture toughness of the material.

## 4. Discussion

### 4.1. Effects of Cooling Rate on Strength

The cooling rate is a critical processing parameter governing the phase transformation kinetics and the resultant mechanical properties of Ti55531 titanium alloy. The volume fraction of the α phase exhibits a monotonic increasing trend as the cooling rate decreases from high to low. When the Ti55531 alloy is cooled from the β single-phase region, rapid cooling (e.g., AC sample) leads the system into a supercooled state, severely restricting the time available for long-range atomic diffusion. Since the β→α transformation in metastable β-titanium alloys is a typical diffusional phase transformation, the nucleation and growth of the α phase are strongly dependent on the redistribution of solute atoms. The insufficient time for diffusion significantly increases the nucleation barrier, suppressing the precipitation of the α phase. Consequently, a considerable portion of the β phase is retained at room temperature in a metastable state. The α phase formed under such a fast-cooling condition typically exhibits a fine acicular morphology, forming a uniform, fine, and interlaced Bs within the β matrix. This structure possesses an extremely high density of α/β interfaces. From the deformation mechanism perspective, these interfaces act as effective barriers to dislocation motion. According to the Hall–Petch relationship (σ_y_ = σ_0_ + k·d^−1/2^, where d represents the effective grain or phase domain size), the decrement in the α phase size (the smaller d value) significantly enhances the strength [34]. Dislocations pile up at the dense α/β interfaces, causing stress concentration, which requires a higher applied stress to initiate slip in adjacent regions, thereby achieving notable interface strengthening [43,44]. Experimental data indicate that the peak tensile strength of air-cooled Ti55531 can reach 1361.4 ± 27.7 MPa, a result primarily attributed to this fine-grain strengthening mechanism.

Conversely, when a slow cooling process is employed, e.g., the 1.0FC sample, the system has ample time for diffusion processes approaching equilibrium. The smaller degree of supercooling allows the α phase to grow preferentially along specific crystallographic orientations, forming a coarse Ws. During this process, not only do the α bundles within Ws become longer and thicker, but the acicular α phase in the Bs also undergoes significant lateral coarsening and lengthening via the Ostwald ripening mechanism. As computed from the EBSD data in Figure 4, the length of the α/β interface decreases from 1706.4 μm in the AC sample to 1226.8 μm in the 2.5FC sample, further to 982.9 μm in the 1.6FC sample, and finally to 781.2 μm in the 1.0FC sample with decreasing cooling rate. This directly leads to a drastic reduction in the number of α/β interfaces per unit volume, significantly weakening the Hall–Petch strengthening effect, and thus resulting in a substantial decrease in the strength. On the other hand, the slow cooling process can essentially be regarded as a prolonged annealing treatment. It promotes the recovery of dislocations and the relaxation of lattice strain introduced during prior thermomechanical processing and phase transformation. The reduction in micro-stresses within both the α and β phases effectively alleviates local stress concentration, delaying the initiation of micro-cracks, thereby substantially improving the plastic deformation capability, which is manifested as a significant increase in elongation in the 1.0FC sample.

It is noteworthy that at an appropriate cooling rate, specifically 2.5 °C/min, the Ti55531 titanium alloy achieves an optimized balance between strength and ductility. Quasi-quantitative analysis indicates that the resultant microstructure typically consists of approximately 13.6% Ws and a predominant Bs. The width of the α bundles in Ws is relatively small (about 0.8 μm), and the width of the acicular α phase in the Bs is only about 140 nm, showing no significant coarsening. This microstructure ingeniously combines the advantages of both structural constituents, i.e., the fine Bs provides a high density of phase interfaces, serving as the primary strengthening component to ensure high strength, while Ws with a moderate proportion and size acts as a relatively softer phase region, capable of coordinating plastic deformation and absorbing crack propagation energy, thereby contributing to higher elongation and fracture toughness. The synergistic interaction between these two structural features is key to the excellent strength–toughness combination of the Ti55531 titanium alloy.

### 4.2. Effects of Cooling Rate on the Fracture Toughness

Fracture toughness progressively increases as the cooling rate decreases, primarily driven by two key factors. First, the reduction in micro-strain plays a crucial role. The XRD analysis shows that the micro-strain of both the α and β phases systematically decreases with lower cooling rates. At higher cooling rates, insufficient atomic diffusion during phase transformation can cause lattice distortion and dislocation accumulation, leading to elevated internal stress (manifested as higher micro-strain). These internal stresses serve as potential crack initiation sites, promoting crack propagation under loading and consequently diminishing material toughness. In contrast, lower cooling rates allow sufficient time for atomic diffusion and lattice relaxation, enabling effective dislocation recovery and micro-strain reduction. This significantly alleviates stress concentration within the material, suppressing both crack initiation and propagation, thereby enhancing fracture toughness.

**Figure 9:**
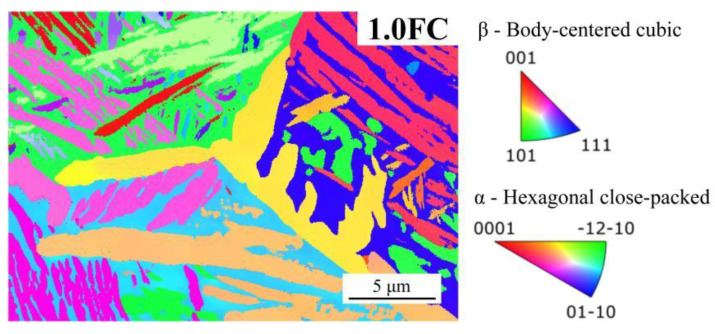
The orientation distribution map of α and β phases in the 1.0FC sample. The corresponding phase map is shown in Figure 4d.

The second critical factor is the significant influence of the cooling rate on both the volume fraction and the microstructure of Ws. This influence manifests primarily through two complementary toughening mechanisms: (1) the inherent plastic deformation capability of the α bundles in Ws, (2) the alteration of the crack propagation path induced by the size and crystallographic orientation of the α bundles. Firstly, Ws exhibits superior plastic compatibility compared to Bs, as shown in Figure 8. The α bundles within Ws typically possess a larger cross-sectional width, which provides more favorable conditions for dislocation glide within them. When a propagating crack encounters an α bundle, the high-stress field at the crack tip can induce significant plastic deformation within the α bundle [32]. This deformation helps to relax the stress concentration, thereby dissipating a substantial amount of fracture energy. In contrast, the fine and randomly oriented acicular α phase in Bs offers limited plastic deformation capacity due to its small size, which severely restricts dislocation motion. Consequently, an increase in the fraction of Ws and the coarsening of its α bundles directly enhance the energy absorption capability within the crack-tip plastic zone. Secondly, with a decreasing cooling rate, not only does the fraction of Ws increase, but the length and width of the α bundles also increase significantly. From the fracture mechanics perspective, the coarsening of the α bundles significantly impacts the effective crack propagation path length. When a crack attempts to penetrate a coarse α bundle, it is forced to travel a longer distance through the relatively softer α lamellae [38] interior or along the α/β interface. This extended path requires greater energy expenditure, thereby significantly increasing the resistance to crack propagation. Furthermore, under sufficiently slow cooling conditions (e.g., in the 1.0FC sample), the α bundles are no longer isolated. Instead, they interweave and connect to form a continuous, interlaced three-dimensional network structure (Figure 3d). Crucially, EBSD analysis reveals significant crystallographic orientation differences between α bundles in adjacent regions, as shown in Figure 9. When a crack encounters this interlaced Ws during propagation, it tends to alternately traverse α bundles with different orientations or propagate along their interfaces (Figure 8(d_2_)). This behavior causes a drastic deflection in the macroscopic crack path, resulting in a characteristic zigzag or serrated trajectory. This path tortuosity similarly increases the effective distance of crack propagation, necessitating greater energy consumption. Therefore, Ws, whose characteristics are precisely tailored by the cooling rate, serves as the core microstructural feature responsible for the significant enhancement of fracture toughness in Ti55531 titanium alloy.

## 5. Conclusions

This study systematically investigates the effects of the cooling rate during β annealing on the microstructure and mechanical properties of Ti55531 titanium alloy. The principal findings are summarized as follows:

(1) As the cooling rate decreases from air cooling to 1.0 °C/min, the α phase fraction increases progressively from 52.67 wt% to 78.24 wt%, accompanied by a gradual reduction in micro-strain within both α and β phases. Lower cooling rates promote the formation and coarsening of Ws, whereas rapid cooling predominantly yields a fine Bs.
(2) Tensile strength decreases with slower cooling, declining from 1361.4 ± 27.7 MPa to 1156.1 ± 25.2 MPa, while elongation and fracture toughness are significantly improved. Notably, a balanced microstructure comprising approximately 13.6% Ws and 86.3% Bs is achieved at a cooling rate of 2.5 °C/min, which exhibits an excellent strength–toughness synergy, with tensile strength of 1252 ± 22.2 MPa, elongation of 9%, and fracture toughness of 84 ± 4 MPa·m^1/2^.
(3) The enhanced fracture toughness is attributed to two complementary mechanisms: (i) reduced micro-strain alleviates stress concentration and suppresses crack initiation; (ii) a coarsened and interlaced Ws effectively deflects crack paths, increases crack propagation length, and enhances energy absorption through plastic deformation within α bundles.

These findings offer a feasible microstructural design strategy to overcome the conventional strength–toughness trade-off in high-strength titanium alloys, providing valuable guidance for the heat treatment optimization of Ti55531 alloy components in aerospace applications.

## Figures and Tables

**Table 1:** Chemical composition of the Ti55531 titanium alloy (mass fraction, %).

Sampling Location	Elemental Content/wt%										
	Al	Mo	V	Cr	Zr	Fe	Si	O	N	C	H
Top	4.79	5.30	5.35	2.86	1.04	0.34	0.015	0.14	0.0066	0.012	0.0027
Bottom	4.77	5.27	5.33	2.85	1.05	0.34	0.015	0.14	0.0053	0.012	0.0026

**Table 2:** The statistical lattice parameters, phase content and micro-strain of α and β phases under different cooling rates.

Sample No.	Lattice Parameter/Å			Phase Content/%		Micro-Strain/%	
	α		β	α	β	α	β
	a = b	c	a = b = c				
AC	2.9303	4.6749	3.2094	53.4 ± 1.0	46.6 ± 1.0	0.336	0.384
2.5FC	2.9287	4.6758	3.2094	59.1 ± 0.3	40.9 ± 0.3	0.282	0.262
1.6FC	2.9286	4.6759	3.2085	69.6 ± 1.5	30.4 ± 1.5	0.272	0.266
1.0FC	2.9290	4.6774	3.2100	77.6 ± 1.0	22.4 ± 1.0	0.235	0.258

**Table 3:** The statistical fraction of Ws and Bs (MIPAR analysis).

Sample No.	Fraction/%	
	Ws	Bs
AC	7.2	92.8
2.5FC	13.6	86.3
1.6FC	27.1	72.9
1.0FC	46.3	53.7

## Data Availability

The original contributions presented in this study are included in the article. Further inquiries can be directed to the corresponding authors.

## References

[1] Leyens C., Peters M. (2003). Titanium and Titanium Alloys-Fundamentals and Applications.

[2] Najafizadeh M., Yazdi S., Bozorg M., Ghasempour-Mouziraji M., Hosseinzadeh M., Zarrabian M., Cavaliere P. (2024). Classification and applications of titanium and its alloys: A review. J. Alloys Compd. Commun..

[3] Kolli R., Arun D. (2018). A review of metastable beta titanium alloys. Metals.

[4] Jones N.G., Dashwood R.J., Dye D., Jackson M. (2008). Thermomechanical processing of Ti-5Al-5Mo-5V-3Cr. Mater. Sci. Eng. A.

[5] Boyer R.R. (1996). An overview on the use of titanium in the aerospace industry. Mater. Sci. Eng. A.

[6] Liu Q.M., Zhang Z.H., Liu S.F., Yang H.Y. (2015). Application and development of titanium alloy in aerospace and military hardware. J. Iron Steel Res..

[7] Beijing Institute of Aeronautical Materials (2013). Materials Technology of Aeronautics.

[8] Yang D.Y., Fu Y.Y., Hui S.X., Ye W.J., Yu Y., Liang E.Q. (2011). Research and application of high strength and high toughness titanium alloys. Chin. J. Rare Met..

[9] Gupta A., Khatirkar R., Singh J. (2022). A review of microstructure and texture evolution during plastic deformation and heat treatment of β-Ti alloys. J. Alloys Compd..

[10] Jiang X.Q., Shi X.Q., Fan X.G., Li Q. (2019). Formation of large size precipitate-free zones in β annealing of the near-β Ti-55531 titanium alloy. Metals.

[11] Liu Z., Deng T.S., Ai R.Y., Yang Y.C., Yuan Y.P., Chen W., He W.H., Li W.R., Xiao W.L. (2024). Microstructure and properties of Ti55531 alloy subjected to deep cryogenic treatment. J. Mater. Res. Technol..

[12] Liu W., Deng H., Chen H.Q., Zhou L., Zuo H.S., Xu P., Qiu W., Chen L., Wei Y., Xia Z. (2022). Ti-5Al-5V-5Mo-3Cr-1Zr (Ti-55531) alloy with excellent mechanical properties fabricated by spark plasma sintering combined with in-situ aging. Mater. Sci. Eng. A.

[13] Ren L., Xiao W.L., Han W.Z., Ma C.L., Zhou L. (2018). Influence of duplex ageing on secondary α precipitates and mechanical properties of the near β-Ti alloy Ti-55531. Mater. Charact..

[14] Cao C.X. (2008). One generation of material technology, one generation of large aircraft. Acta Aeronaut. Astronaut. Sin..

[15] Chen F.W., Xu G.L., Zhang X.Y., Zhou K.C. (2017). Isothermal kinetics of β↔a transformation in Ti-55531 alloy influenced by phase composition and microstructure. Mater. Des..

[16] Warchomicka F., Poletti C., Stockinger M. (2011). Study of the hot deformation behaviour in Ti-5Al-5Mo-5V-3Cr-1Zr. Mater. Sci. Eng. A.

[17] Chang X.S., Qi Y.S., Chen X.L., Xie Z.Y., Zhang P., Chen G. (2023). Extra work hardening and activation of softening mechanisms induced by α-β interactions of a metastable-β Ti alloy during subtransus processing. Mater. Sci. Eng. A.

[18] Ding H.L., Wang L.L., Yuan L.K., Lin X., Huang W.D. (2024). Effect of trace B on microstructure and mechanical properties of additive manufactured near β titanium alloy Ti55531. Adv. Eng. Mater..

[19] Wang Q.R., Sha A.X., Huang L.J., Li X.W. (2014). Influence of heat treatment process on microstructure and mechanical properties of Ti-55531 alloy. Titan. Ind. Prog..

[20] Tan C.S., Yang T., He J.H., Dang Q., Lu H.P., Wen L.X., Zhang G.J. (2025). Influence of electropulsing treatment and step-quench process on the gradient structure evolution of Ti55531 alloy. J. Mater. Eng. Perform..

[21] Zhang C., Yu H., Liu X.Y., Xu W., Lu X. (2025). Powder metallurgy combined with β single-phase zone hot deformation for microstructure and mechanical properties regulation of ultra-high strength and toughness Ti-55531 titanium alloy. J. Alloys Compd..

[22] Huang C.W., Zhao Y.Q., Xin S.W., Zhou W., Li Q., Zeng W.D. (2017). Effect of microstructure on tensile properties of Ti-5A1-5Mo-5V-3Cr-1Zr alloy. J. Alloys Compd..

[23] Huang C.W., Zhao Y.Q., Xin S.W., Zhou W., Li Q., Zeng W.D., Tan C.S. (2017). High cycle fatigue behavior of Ti-5Al-5Mo-5V-3Cr-1Zr titanium alloy with bimodal microstructure. J. Alloys Compd..

[24] Xu Z.L., Huang C.W., Tan C.S., Wan M.P., Zhao Y.Q., Ye J.Q., Zeng W.D. (2021). Influence of microstructure on cyclic deformation response and micromechanics of Ti-55531 alloy. Mater. Sci. Eng. A.

[25] Wen X., Xin R.L., Huang C.W., Xin S.W. (2025). Transformation twins enable high strength-ductility synergy in a lamellar Ti-55531 alloy: Variant selection and deformation mechanisms. J. Alloys Compd..

[26] Li Z.Y., Wu G.Q., Huang Z. (2018). Relationships between microstructure and mechanical properties of Ti-5A1-5Mo-5V-3Cr-1Zr alloy. Mater. Res. Express.

[27] Li P., Sun Q.Y., Xiao L., Sun J. (2020). Tuning the morphology of Ti-5Al-5Mo-5V-3Cr-1Zr alloy: From brittle to ductile fracture. Mater. Sci. Eng. A.

[28] Wu C., Zhan M. (2019). Microstructural evolution, mechanical properties and fracture toughness of near β titanium alloy during different solution plus aging heat treatments. J. Alloys Compd..

[29] Chen G., Zhou X.X., Sun Z.Y., Jia P., Su H., Liu L.H., Qi Y.S., Ye J.Q., Yu Z., Chang X.S. (2025). Spheroidization and subsequent homogenization of lamellar α phase in metastable-β Ti alloys during severe plastic deformation. J. Alloys Compd..

[30] Ding H.L., Wang L.L., Lin X., Xue A.T., Yuan L.K., Dang M.J., Huang W.D. (2022). Simultaneously enhancing strength and toughness of heat-treated near β titanium alloy fabricated by laser-directed energy deposition. Mater. Sci. Eng. A.

[31] Liu C.M., Yu L., Zhang A.L., Tian X.J., Liu D., Ma S.Y. (2016). Beta heat treatment of laser melting deposited high strength near β titanium alloy. Mater. Sci. Eng. A.

[32] Gil F.J., Ginebra M.P., Manero J.M., Planell J.A. (2001). Formation of α-Widmanstätten structure: Effects of grain size and cooling rate on the Widmanstätten morphologies and on the mechanical properties in Ti6Al4V alloy. J. Alloys Compd..

[33] Xu J.W., Zeng W.D., Zhao Y.W., Sun X.H., Du Z.L. (2016). Influence of cooling rate following heat treatment on microstructure and phase transformation for a two-phase alloy. J. Alloys Compd..

[34] Hao M., Wang D., Wang Y.L., Zhang T.L., Li P., Guo Y.N., Zheng Y.F., Sun Q.Y., Wang Y.Z. (2024). Heterogeneous precipitate microstructure design in β-Ti alloys by regulating the cooling rate. Acta Mater..

[35] Welsch G., Boyer R., Collings E.W. (1993). Materials Properties Handbook: Titanium Alloys.

[36] Zhou Y.T., Zhou J., Shu Q., Li S.S., Gongye F.J., Long S., Deng H.P. (2020). Deformation mechanism and constitutive consideration for Ti-5Al-5Mo-5V-3Cr-1Zr alloy compressed at elevated temperatures. J. Mater. Eng. Perform..

[37] Yang Y.C., Deng T.S., Liu Z., Liu H., Yuan Y.P., Chen W. (2025). Deformation behavior and microstructure evolution of high-strength and -toughness Ti55531 titanium alloy. Metals.

[38] Huang C.W., Zhao Y.Q., Xin S.W., Tan C.S., Zhou W., Li Q., Zeng W.D. (2017). High cycle fatigue behavior of Ti-5Al-5Mo-5V-3Cr-1Zr titanium alloy with lamellar microstructure. Mater. Sci. Eng. A.

[39] Wang G.N., Zhang X.Y., Li Z.Y., Zhou K.C. (2014). Phase transformation of Ti55531 alloy during continuous heating process. Chin. J. Nonferrous Met..

[40] Pan H. (2016). Influence of Microstructure on Mechanical Property and Hot Deformation Behavior of High Strength-Toughness Ti-55531 Alloy. Master’s Thesis.

[41] (2021). Metallic Materials—Tensile Testing—Part 1: Method of Test at Room Temperature.

[42] (2007). Metallic Materials—Determination of Plane-Strain Fracture Toughness.

[43] Liu Y., Lim S.C., Ding C., Huang A.J., Weyland M. (2022). Unravelling the competitive effect of microstructural features on the fracture toughness and tensile properties of near beta titanium alloys. J. Mater. Sci. Technol..

[44] Zhu W.G., Lei J., Tan C.S., Sun Q.Y., Chen W., Xiao L., Sun J. (2019). A novel high-strength β-Ti alloy with hierarchical distribution of α-phase: The superior combination of strength and ductility. Mater. Des..

